# Tension pneumoperitoneum combined with CO_2_ gas embolism during peroral endoscopic myotomy: a case report and review of literature

**DOI:** 10.3389/fmed.2026.1817498

**Published:** 2026-05-15

**Authors:** Shui Yu, Xiaofeng Hu, Jingyan Xu, Qin Wang, Jingxiong Zhang, Kai Sun, Xiaoxia Zhou

**Affiliations:** Department of Anesthesiology, The Second Affiliated Hospital of Zhejiang University School of Medicine, Hangzhou, China

**Keywords:** achalasia, gas embolism, insufflation-related adverse events, peroral endoscopic myotomy, tension pneumoperitoneum

## Abstract

**Background:**

Peroral endoscopic myotomy (POEM) is a safe, effective, and minimally invasive treatment for achalasia. While continuous carbon dioxide (CO_2_) insufflation during POEM may lead to various insufflation-related adverse events, the occurrence of gas embolism remains an exceedingly rare complication. This report describes a case of tension pneumoperitoneum complicated by carbon dioxide gas embolism during POEM.

**Case:**

A 51-year-old man was diagnosed with achalasia and underwent POEM. Intraoperatively, the end-tidal carbon dioxide (PetCO_2_) continuously increased to 70 mmHg, accompanied by clinical manifestations of tension pneumoperitoneum. The peak inspiratory pressure (PIP) rose abruptly from 16 to 40 cmH_2_O, alongside significant subcutaneous emphysema and abdominal distension. Vital signs gradually stabilized following percutaneous abdominal needle decompression (PND). However, when the patient’s position was changed from left lateral decubitus to supine, PetCO_2_ suddenly dropped from 68 to 53 mmHg, and peripheral oxygen saturation (SpO_2_) declined to 90%, accompanied by frequent ventricular premature beats. A characteristic “mill-wheel” murmur was audible over the precordium, leading to a high suspicion of CO_2_ gas embolism. Immediate interventions, including oxygen administration, positional adjustment, and supportive care, resulted in rapid clinical improvement. The patient was subsequently transferred to the intensive care unit (ICU) for further management and was successfully discharged on postoperative day 6 without additional complications.

**Conclusion:**

During POEM, elevated intra-abdominal pressure resulting from tension pneumoperitoneum represents a significant risk factor for gas embolism. The simultaneous occurrence of multiple complications under general anesthesia further increases diagnostic complexity, as clinical signs may be masked or overlapping. Therefore, close intraoperative monitoring, early recognition of pathognomonic indicators, and prompt intervention are essential to ensure patient safety.

## Introduction

Achalasia is an esophageal motility disorder characterized by impaired esophageal peristalsis and dysfunction of the lower esophageal sphincter (LES) (1). The European Society of Gastrointestinal Endoscopy recommends peroral endoscopic myotomy (POEM) as a first-line treatment for achalasia (2), with reported short-term efficacy rates of 90–100% (3, 4) and a 5-year success rate of approximately 83% (5). Despite these favorable outcomes, continuous carbon dioxide insufflation during POEM can lead to insufflation-related adverse events, including tension pneumoperitoneum, subcutaneous emphysema, pulmonary emphysema, and pneumothorax (6). Previous laparoscopic surgeries have confirmed that elevated intra-abdominal pressure represents a high-risk factor for gas embolism (7). When tension pneumoperitoneum and gas embolism occur concurrently, severe respiratory and circulatory dysfunction may occur. Such combined events are extremely rare during POEM, and clinical reports remain limited. Here, a rare case of tension pneumoperitoneum complicated by carbon dioxide gas embolism during POEM is presented to increase awareness of this potentially life-threatening complication and emphasize the need for vigilance and timely management during the procedure.

## Case report

A 51-year-old man (height 174 cm, weight 85 kg) presented with progressive dysphagia for more than 1 year. Preoperative investigations provided a comprehensive clinical picture: gastroscopy identified substantial food and fluid retention, a constricted cardia, and proximal esophageal dilation. Mediastinal CT imaging corroborated these findings, showing global esophageal enlargement. Barium esophagography further visualized a pathognomonic “bird-beak” narrowing of the distal esophagus, characterized by restricted passage of contrast into the stomach without evidence of mucosal ulceration or space-occupying lesions. Diagnosis was finalized as Type II achalasia via high-resolution manometry (HRM), adhering to the Chicago Classification version 4.0 criteria. With satisfactory cardiopulmonary function confirmed, the patient proceeded to peroral endoscopic myotomy (POEM).

Baseline vital signs were stable: heart rate 72 beats/min, blood pressure 125/79 mmHg, and SpO_2_ 99% on room air. After awake gastroscopy (GIF-Q260J; Olympus, Tokyo, Japan) to aspirate esophageal contents and preoxygenation with 100% oxygen, rapid-sequence induction of anesthesia was performed using intravenous midazolam, alfentanil, etomidate, and rocuronium. Tracheal intubation was successfully achieved and secured. Anesthesia was maintained with a mixture of air and oxygen, continuous infusion of propofol and remifentanil, and intermittent cisatracurium to maintain muscle relaxation. Volume-controlled ventilation was applied with the following settings: FiO_2_ 50%, tidal volume 500 mL, and respiratory rate 12 bpm. During the initial phase of the procedure, vital signs remained stable (heart rate 75 bpm, blood pressure 115/60 mmHg, SpO_2_ 99%), with a peak inspiratory pressure of 16 cmH_2_O and baseline end-tidal CO_2_ (PetCO_2_) of 35 mmHg.

Low-flow carbon dioxide insufflation (approximately 1.2 L/min) was initiated *via* the gastroscope equipped with a transparent distal attachment (D-201-11804; Olympus). The cardia was noted to be narrowed at 41 cm from the incisors, with significant esophageal tortuosity. Following submucosal injection at 30 cm from the incisors, a 2.0-cm longitudinal mucosal incision was made on the posterior esophageal wall using a DualKnife (KD-650 L; Olympus). A submucosal tunnel was then created, extending to the gastric cardia at 46 cm from the incisors.

Subsequently, a full-thickness myotomy with a total length of 8 cm (comprising a 6-cm esophageal myotomy and a 2-cm gastric myotomy) was performed using low-power electrocoagulation. After confirming adequate relaxation of the cardia, hemostasis within the tunnel was ensured using a Coagrasper (FD-411UR; Olympus). The mucosal entry was then closed with seven rotatable repositionable clips (ROCC-D-26-195-C; Micro-Tech, Nanjing, China). The total operative duration was 64 min.

Following esophageal incision and insufflation, PetCO_2_ gradually increased, and ventilatory parameters were adjusted to maintain PetCO_2_ at approximately 50 mmHg. After completion of the esophageal myotomy, PetCO_2_ increased further to 70 mmHg, accompanied by a decrease in SpO_2_ to 85%. At this time, heart rate increased to 102 beats/min, blood pressure (BP) decreased to 98/49 mmHg, and peak inspiratory pressure (PIP) increased abruptly from 16 to 40 cmH_2_O. Switching to 100% oxygen did not improve oxygen saturation. Bilateral breath sounds were symmetric on auscultation, and no airway secretions were detected. Subcutaneous emphysema was observed over the neck and chest, and the abdomen was significantly distended and tense. Suspecting the patient developed a tension pneumoperitoneum, the surgeon promptly clipped the mucosal entry to conclude the procedure and performed percutaneous abdominal needle decompression (PND).

Arterial blood gas analysis under mechanical ventilation with 100% oxygen showed severe respiratory acidosis (pH 7.13, PCO₂ 85 mmHg, PO₂ 149 mmHg, base excess −3.7 mmol/L), with potassium 4.49 mmol/L, lactate 1.3 mmol/L, and hemoglobin 143 g/L. After decompression, SpO₂ gradually improved to 98%, PIP decreased to 19 cmH₂O, and PetCO₂ fell to 68 mmHg. Hemodynamics stabilized. However, upon completion of the procedure, as the patient was repositioned from the left lateral to the supine position, PetCO_2_ suddenly decreased to 53 mmHg, and SpO_2_ again declined to 90%, accompanied by frequent ventricular premature beats. Bilateral breath sounds remained symmetric, but a characteristic “mill-wheel” murmur was audible over the precordium. As abdominal distension had resolved, carbon dioxide gas embolism was suspected in the patient. Emergency support was initiated immediately. The patient was placed in the left lateral (Durant) and Trendelenburg positions, ventilation with 100% oxygen was continued, and 0.1 mg lidocaine was administered for ventricular ectopy. Intravenous fluids were given to support circulation. Bedside transthoracic echocardiography (TTE) was performed simultaneously. Following these interventions, vital signs improved promptly, and sinus rhythm was restored. Due to chest wall emphysema, echocardiographic imaging quality was limited, and no definite intracardiac gas bubbles were visualized. Repeat arterial blood gas analysis showed partial improvement (pH 7.17, PCO₂ 74 mmHg, PO₂ 204.6 mmHg, base excess −3.7 mmol/L, potassium 4.58 mmol/L, lactate 1.3 mmol/L, hemoglobin 142 g/L).

After 45 min of observation with stable vital signs, the patient was transferred to the intensive care unit (ICU) for further management. Emergency chest computed tomography (CT) demonstrated mild bilateral pneumothorax and hydrothorax, dorsal lung atelectasis, and small amounts of mediastinal and right cervicothoracic subcutaneous emphysema. Abdominal CT revealed postoperative changes near the cardia and multiple free gas collections within the abdominal cavity (Supplementary Figure S1).

Axial radiographs showed a large amount of free gas in the abdominal cavity after POEM surgery. Significant mass effect was observed, manifested as compression and medial displacement of the liver and spleen, indicating increased intra-abdominal pressure.

The endotracheal tube was removed the following morning in the ICU. The patient remained alert and hemodynamically stable and was transferred to the general ward later that day. He recovered uneventfully and was discharged on postoperative day 6.

## Discussion

This report describes a rare but life-threatening complication during POEM: tension pneumoperitoneum complicated by CO_2_ gas embolism. POEM has gained wide acceptance due to its efficacy and minimally invasive profile. However, adverse events related to CO₂ insufflation are not uncommon, with reported incidences ranging from 7.5 to 55% (8) and even approaching 100% (9). This underscores the imperative for anesthesiologists to maintain high vigilance for extra-luminal gas syndromes beyond routine ventilation management.

During POEM, continuous monitoring of respiratory and hemodynamic parameters is essential. Maintaining exposure of the upper abdomen is recommended to facilitate early detection of abdominal distension. Pneumoperitoneum may be suspected based on increasing abdominal tension, tympanic percussion, and a rise in PIP, with an increase exceeding 20% of baseline regarded as an important warning sign (10). In this case, PIP increased abruptly from 16 to 40 cmH₂O, accompanied by significant abdominal distension, which together strongly supported the diagnosis of tension pneumoperitoneum. Pathophysiologically, tension pneumoperitoneum compromises pulmonary compliance and compresses the inferior vena cava, thereby reducing cardiac preload and output. In patients with limited cardiopulmonary reserve, such disturbances may rapidly progress to severe hemodynamic instability or even circulatory failure. Notably, given the preservation of symmetric breath sounds and the immediate hemodynamic resolution following PND, it is reasonable to infer that the acute instability was primarily driven by intra-abdominal hypertension rather than the concurrent mild pneumothorax.

Several factors during POEM increase the risk of gas embolism, including unavoidable disruption of the mucosal barrier, continuous CO₂ insufflation, and the development of tension pneumoperitoneum (11). When intraluminal or intra-abdominal pressure exceeds venous pressure, CO_2_ may enter the circulation through injured vessels, resulting in acute gas embolism. In this case, after decompression of the pneumoperitoneum, a sudden decrease in PetCO₂, recurrent oxygen desaturation, frequent ventricular premature beats, and the presence of a precordial “mill-wheel” murmur occurred when the patient was repositioned from the left lateral to the supine position. These findings were highly suggestive of CO_2_ embolism. This temporal association indicates that changes in body position may serve as a trigger for the clinical manifestation of occult gas embolism. A plausible mechanism is that positional changes allow gas sequestered within the venous system or right heart to migrate into the pulmonary circulation *via* the right ventricular outflow tract, causing acute pulmonary vascular obstruction. It is noteworthy that the precipitous drop in PetCO_2_ likely reflects an acute increase in alveolar dead-space ventilation—a hallmark of gas embolism—rather than simple restrictive ventilatory impairment associated with tension pneumoperitoneum.

Reports of gas embolism during POEM remain exceedingly rare. Available evidence indicates that the mortality associated with post-endoscopic gas embolism ranges from 12 to 30% (12), and may reach as high as 57.1% in severe cases (11). While previously documented in modalities such as ERCP and ESD (13, 14), its occurrence during POEM has seldom been reported, potentially due to the frequent masking of symptoms under general anesthesia, making early identification challenging. The severity of gas embolism depends largely on both the rate and volume of gas entering the circulation (15). Because presenting signs and symptoms are frequently nonspecific, intraoperative diagnosis requires a high index of suspicion. Classical descriptions include cyanosis, a precordial “mill-wheel” murmur, abrupt decreases in PetCO₂, hypotension, and tachycardia, although these features are not consistently present. A retrospective analysis of seven cases of gas embolism during laparoscopic surgery showed that less than half exhibited a characteristic murmur; more common findings included arrhythmias and neurological symptoms (16).

Several monitoring methods may facilitate intraoperative detection. Continuous PetCO₂ monitoring is considered as a particularly sensitive and non-invasive tool, and a sudden decrease in PetCO₂ in intubated patients may represent one of the earliest indicators of gas embolism (17). Transesophageal echocardiography (TEE) is considered the most sensitive diagnostic technique, capable of detecting gas bubbles as small as 20–50 μm, particularly within the right ventricular outflow tract or pulmonary artery. However, despite its better sensitivity, TTE is more commonly used in clinical practice because of its accessibility and noninvasive nature. In this case, image quality was limited by subcutaneous emphysema, and typical echocardiographic features were not observed, potentially due to rapid CO_2_ absorption. Furthermore, although CT is sensitive for detecting intravascular gas, its sensitivity and specificity for right ventricular overload are relatively low (81 and 47%, respectively), limiting its utility in critically ill patients (18). For patients suspected of having systemic gas embolism, CT scans are perhaps best reserved for post-stabilization assessment to evaluate gas distribution (19).

Given that intra-abdominal hypertension resulting from tension pneumoperitoneum represents a major risk factor for gas embolism, several preventive strategies should be considered to reduce its occurrence. First, air should be avoided as an insufflation medium (20, 21). Second, minimizing total insufflation volume is critical. Unlike laparoscopic surgery, POEM typically lacks automatic regulation of CO_2_ flow to maintain constant intraluminal pressure; therefore, endoscopists should use the lowest effective flow rate (6, 22). If tension pneumoperitoneum develops intraoperatively and abdominal pressure exceeds safe limits, the procedure should be suspended until intra-abdominal pressure normalizes. Close multidisciplinary collaboration between endoscopists and anesthesiologists is essential, and prompt PND should be performed to relieve abdominal hypertension (23). For enhanced safety, ultrasound-guided puncture has also been recommended by some investigators (8).

Once gas embolism is highly suspected, immediate coordinated action by the surgical and anesthesia teams is required. It is generally advisable to terminate the procedure without delay, hemodynamic and respiratory stability must be restored, and 100% oxygen ventilation must be initiated. Placement in the left lateral decubitus (Durant) position combined with Trendelenburg positioning helps direct gas toward the cardiac apex and away from the right ventricular outflow tract, while also reducing the risk of cerebral embolization. Moderate fluid administration can increase venous pressure and support blood pressure. Hyperbaric oxygen therapy (HBO), considered first-line treatment for severe gas embolism, with optimal clinical efficacy observed when initiated within a 6–8 h therapeutic window (24). Mechanistically, HBO reduces bubble volume, promotes gas resorption, and increases arterial oxygen content, thereby reducing ischemic injury, particularly to the brain. Crucially, a rigorous clinical assessment for concurrent pneumothorax or other pulmonary pathologies is mandatory prior to HBO; as the ambient pressure during hyperbaric exposure can exacerbate barotrauma, potentially leading to catastrophic clinical outcomes (17).

## Conclusion

In summary, in addition to insufflation-related adverse events, sudden intraoperative hemodynamic instability during POEM should raise suspicion for the rare but potentially fatal complication of gas embolism. The coexistence of multiple complications significantly increases diagnostic and therapeutic complexity. This underscores the need for anesthesiologists and endoscopists to monitor, promptly recognize, and implement crisis management protocols to ensure patient safety.

## Patient perspective

We obtained the patient’s written informed consent to disclose his case. In the form the patient(s) has/have given his/her/their consent for his/her/their images and other clinical information to be reported in the journal. The patients understand that their names and initials will not be published and due efforts will be made to conceal their identity.

## Data Availability

The original contributions presented in the study are included in the article/Supplementary material, further inquiries can be directed to the corresponding author.

## Supplementary material

SUPPLEMENTARY FIGURE S1 Abdominal CT scan showed signs of suspected pneumoperitoneum. The red arrow demonstrated a crescentic focus of free intraperitoneal air anterior to the liver.
