# Supplementary figures and images for: Tension pneumoperitoneum combined with CO2 gas embolism during peroral endoscopic myotomy: a case report and review of literature
Source: Front Med (Lausanne). 2026 May 15;13:1817498. doi: 10.3389/fmed.2026.1817498 (PMC13219238; doi:10.3389/fmed.2026.1817498)

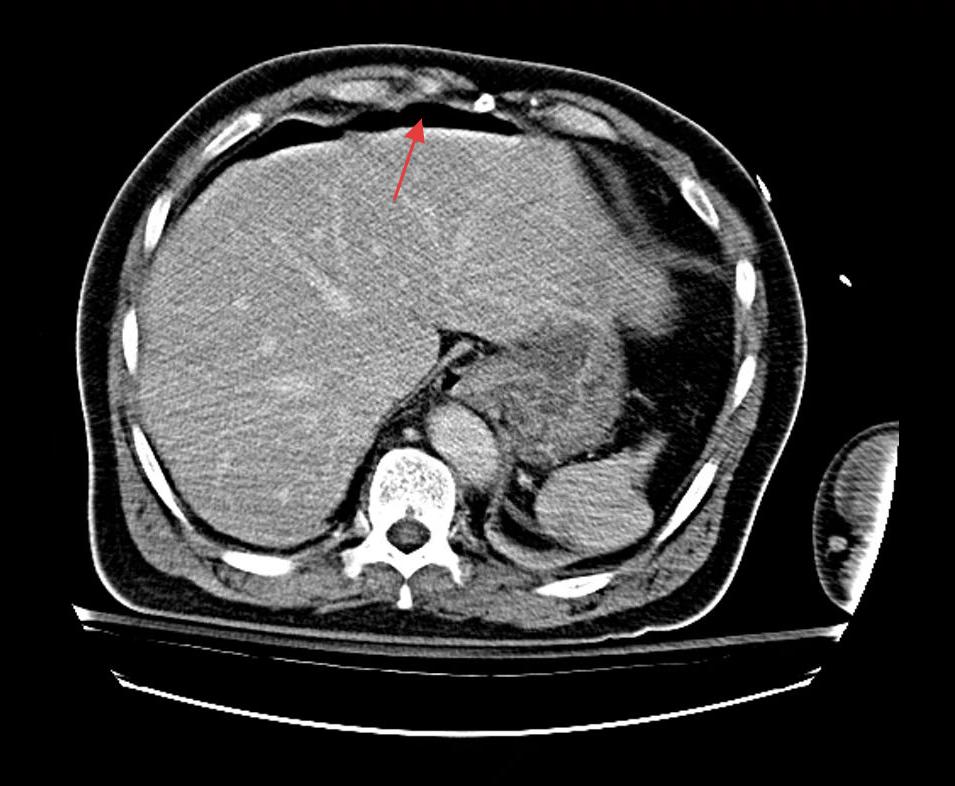

Supplement: SUPPLEMENTARY FIGURE S1 — Abdominal CT scan showed signs of suspected pneumoperitoneum. The red arrow demonstrated a crescentic focus of free intraperitoneal air anterior to the liver. [file Image_1.jpeg]
